# The evolution of Kenya’s animal health surveillance system and its potential for efficient detection of zoonoses

**DOI:** 10.3389/fvets.2024.1379907

**Published:** 2024-06-20

**Authors:** Samuel Kahariri, S. M. Thumbi, Bernard Bett, Marianne W. Mureithi, Nazaria Nyaga, Allan Ogendo, Mathew Muturi, Lian Francesca Thomas

**Affiliations:** ^1^Directorate of Veterinary Services, Nairobi, Kenya; ^2^International Livestock Research Institute, Nairobi, Kenya; ^3^Department of Medical Microbiology and Immunology, Faculty of Health Sciences, University of Nairobi, Nairobi, Kenya; ^4^Centre for Epidemiological Modelling and Analysis, Institute of Tropical and Infectious Diseases, University of Nairobi, Nairobi, Kenya; ^5^Institute of Immunology and Infection Research, University of Edinburgh, Edinburgh, United Kingdom; ^6^Paul G. Allen School for Global Health, Washington State University, Pullman, WA, United States; ^7^County Directorate of Veterinary Services, Kajiado, Kenya; ^8^County Directorate of Veterinary Services, Busia, Kenya; ^9^Institute of Infection Veterinary and Ecological Sciences, University of Liverpool, Neston, United Kingdom

**Keywords:** surveillance, one health, early warning, animal health, integration

## Abstract

**Introduction:**

Animal health surveillance systems in Kenya have undergone significant changes and faced various challenges throughout the years.

**Methods:**

In this article, we present a comprehensive overview of the Kenya animal health surveillance system (1944 to 2024), based on a review of archived documents, a scoping literature review, and an examination of past surveillance assessments and evaluation reports.

**Results:**

The review of archived documents revealed key historical events that have shaped the surveillance system. These include the establishment of the Directorate of Veterinary Services in 1895, advancements in livestock farming, the implementation of mandatory disease control interventions in 1944, the growth of veterinary services from a section to a ministry in 1954, the disruption caused by the Mau Mau insurrection from 1952 to 1954, which led to the temporary halt of agriculture in certain regions until 1955, the transition of veterinary clinical services from public to private, and the progressive privatization plan for veterinary services starting in 1976. Additionally, we highlight the development of electronic surveillance from 2003 to 2024. The scoping literature review, assessments and evaluation reports uncovered several strengths and weaknesses of the surveillance system. Among the strengths are a robust legislative framework, the adoption of technology in surveillance practices, the existence of a formal intersectoral coordination platform, the implementation of syndromic, sentinel, and community-based surveillance methods, and the presence of a feedback mechanism. On the other hand, the system’s weaknesses include the inadequate implementation of strategies and enforcement of laws, the lack of standard case definitions for priority diseases, underutilization of laboratory services, the absence of formal mechanisms for data sharing across sectors, insufficient resources for surveillance and response, limited integration of surveillance and laboratory systems, inadequate involvement of private actors and communities in disease surveillance, and the absence of a direct supervisory role between the national and county veterinary services.

**Discussion and recommendations:**

To establish an effective early warning system, we propose the integration of surveillance systems and the establishment of formal data sharing mechanisms. Furthermore, we recommend enhancing technological advancements and adopting artificial intelligence in surveillance practices, as well as implementing risk-based surveillance to optimize the allocation of surveillance resources.

## 1 Introduction

Emerging Infectious Diseases (EID) pose a global challenge to economies and public health (1). The majority (60%) of these EID events are zoonotic, with nearly three out of every four events (72%) originating from wildlife (1–3). The drivers of emergence are multifaceted including environmental, ecological, and socio-economic changes that facilitate novel or increased contact between wildlife, livestock and humans leading to the transmission of pathogens between hosts (1). Therefore, One Health (OH) approaches that involve collaboration between animal, human, and environmental sectors are critical for understanding the emergence of zoonotic infections, their early detection before spilling over to humans, and for timely response to control their spread and impact (4).

Early warning systems for health-related events should provide timely information and be sensitive enough to capture and analyze any unusual patterns in the occurrence of health events, animal diseases, and diseases transmissible between humans and animals (zoonoses) for prompt epidemiological response actions (5). To be useful for early warning systems, the surveillance system ought to employ an OH approach, to foster collaborations and preparedness across the sectors (6, 7) and align with the current global focus on deep prevention which emphasizes on mid-stream and upstream prevention of zoonoses (8). The system should utilize real-time digital tools in data collection and reporting (9, 10), utilize risk assessment and modeling techniques to enhance forecasting of events of public health and economic importance (10) and strengthen laboratory capacity for both human and animal cases (10–12). The surveillance system should also benefit from syndromic surveillance (13), operate within a robust legal framework (10) and financing mechanism (14), and continuously build capacity of surveillance officers across the sectors at all levels of government (10, 15).

Animal disease surveillance safeguards the health and welfare of animals and public health, ensures the safety of foods of animal origin, and provides quality assurance for trade in animals and animal products. Importantly animal health data also provides useful information for the timely detection of potential hazards and ensuring appropriate actions can be taken to safeguard public health and decision-making and priority setting for control measures (16).

Kenya, like the rest of the East Africa region, carries a large burden or is at risk of multiple EID of zoonotic origin including Rift Valley Fever (RVF), dengue, and yellow fever. It also experiences endemic zoonoses such as anthrax, rabies, brucellosis, trypanosomiasis, bovine tuberculosis, cysticercosis, leishmaniasis, echinococcosis, and other transboundary animal diseases (17). Surveillance of these diseases in the animal population may support forecasting of disease risks to humans (18).

Animal health surveillance systems in Kenya have evolved based on the changing needs. This evolution provides important lessons that are critical for the development and maintenance of a robust system. In this manuscript, we review the evolution of animal health surveillance in Kenya over the last 80 years, examine the evaluations undertaken on Kenya’s animal health surveillance system, and provide a perspective on the opportunities and challenges of the current Kenyan animal health surveillance system in providing early warning for infectious disease threats of zoonotic origin.

## 2 Methods

This manuscript provides a synthesis of Animal Health (AH) surveillance systems and tools in Kenya from 1944 to 2024 drawn from a three-stage review: stage one reviewed documents held at the Directorate of Veterinary Services (DVS), specifically: transcripts from historical government reports, disease control strategies, and reports of consultative forums involving national and county surveillance officers generated during the Kenya Animal Bio-surveillance System (KABS) rollout and training workshops (19). Secondly, we conducted a scoping review of the literature. The first author identified and reviewed articles on animal health surveillance in Kenya during the study period using the PubMed database. The search syntax used was “((Kenya[Title]) AND (surveillance[Title/Abstract])) AND (disease[Title/Abstract]).” We included articles that contained information relevant to Kenya animal health surveillance system and excluded articles that were not related to animal health events or surveillance, as well as articles that did not contain any information about Kenya animal health surveillance. The selected articles were then reviewed based on specific attributes of a surveillance system including usefulness, simplicity, flexibility, acceptability, positive predictive value, representativeness, timeliness, and collaborations (20). The first author extracted relevant data into a Word document categorizing it according to the attribute it addressed. This data was then then refined through consensus with the other authors.

**Figure 1:**
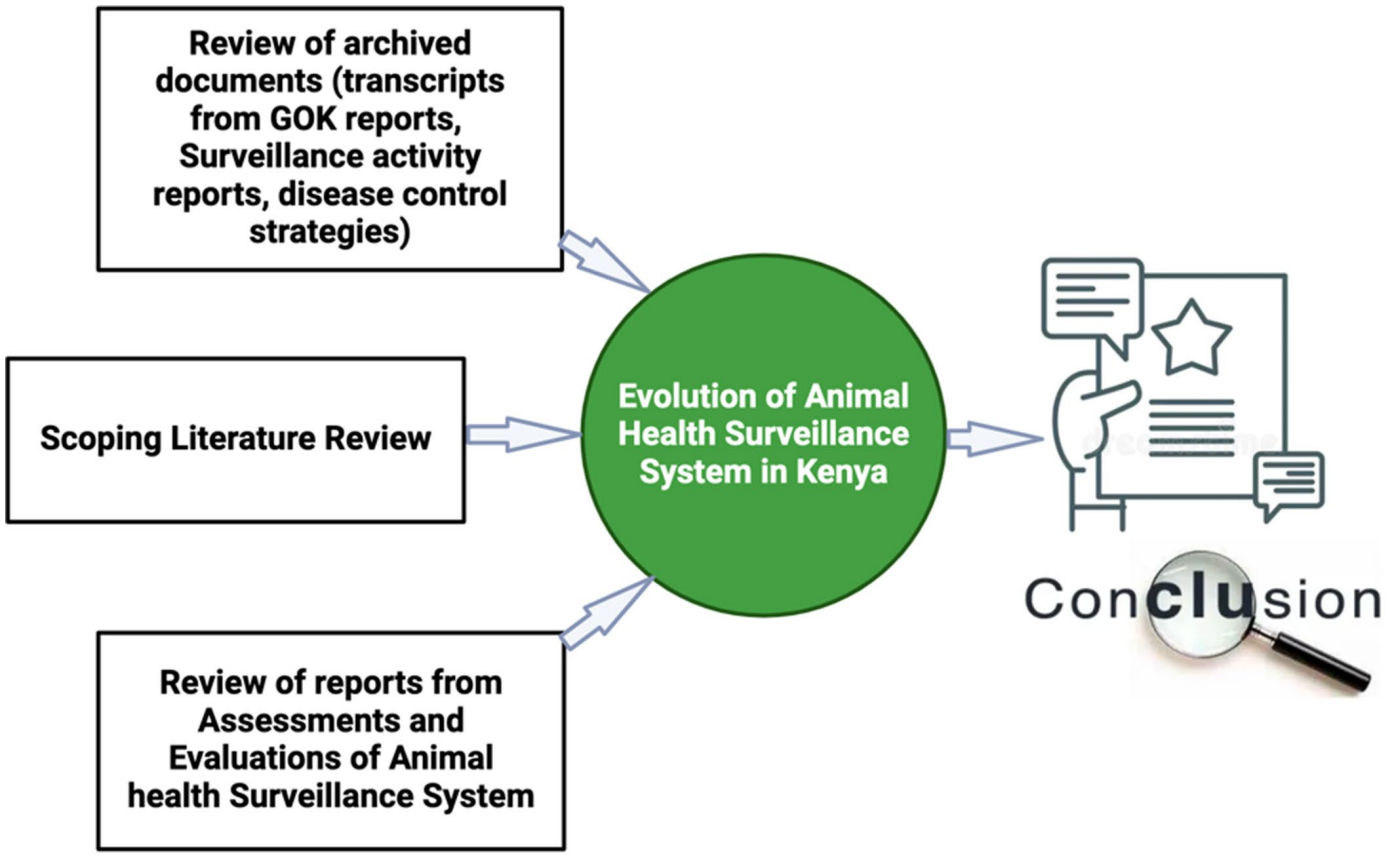
Summary of the approach used to collect information used to document the evolution of animal surveillance systems in Kenya for the period of 1944 – to-date.

Finally, we reviewed reports on assessments and evaluations of animal health surveillance systems undertaken in Kenya for the period between 2011 to 2023. Additionally, we present the perspective of the authors, who have diverse backgrounds ranging from governmental officers involved in developing and utilizing surveillance tools to researchers and academics who have implemented surveillance programs across various regions in Kenya. The information gathered in this study was utilized to enhance our understanding and document the evolution of animal health surveillance in Kenya, starting from 1944 up to the present. Furthermore, it provides insights into the strengths, identified gaps, and potential solutions, as well as assesses the reporting rates and trends over time. Lastly, the retrospective analysis of the system against key surveillance attributes will inform its capacity for early detection of zoonotic diseases and offer valuable lessons for strengthening the system. The summary of the process is outlined in the flow diagram in Figure 1.

## 3 Results

The review of archived documentation captured 20 records. The documents are provided for in the supplementary materials. Key information regarding surveillance was extracted and included in our record. Our scoping review identified 21 relevant articles from which key findings were extracted having direct or indirect implications on the surveillance systems (21–40).

**Figure 2:**
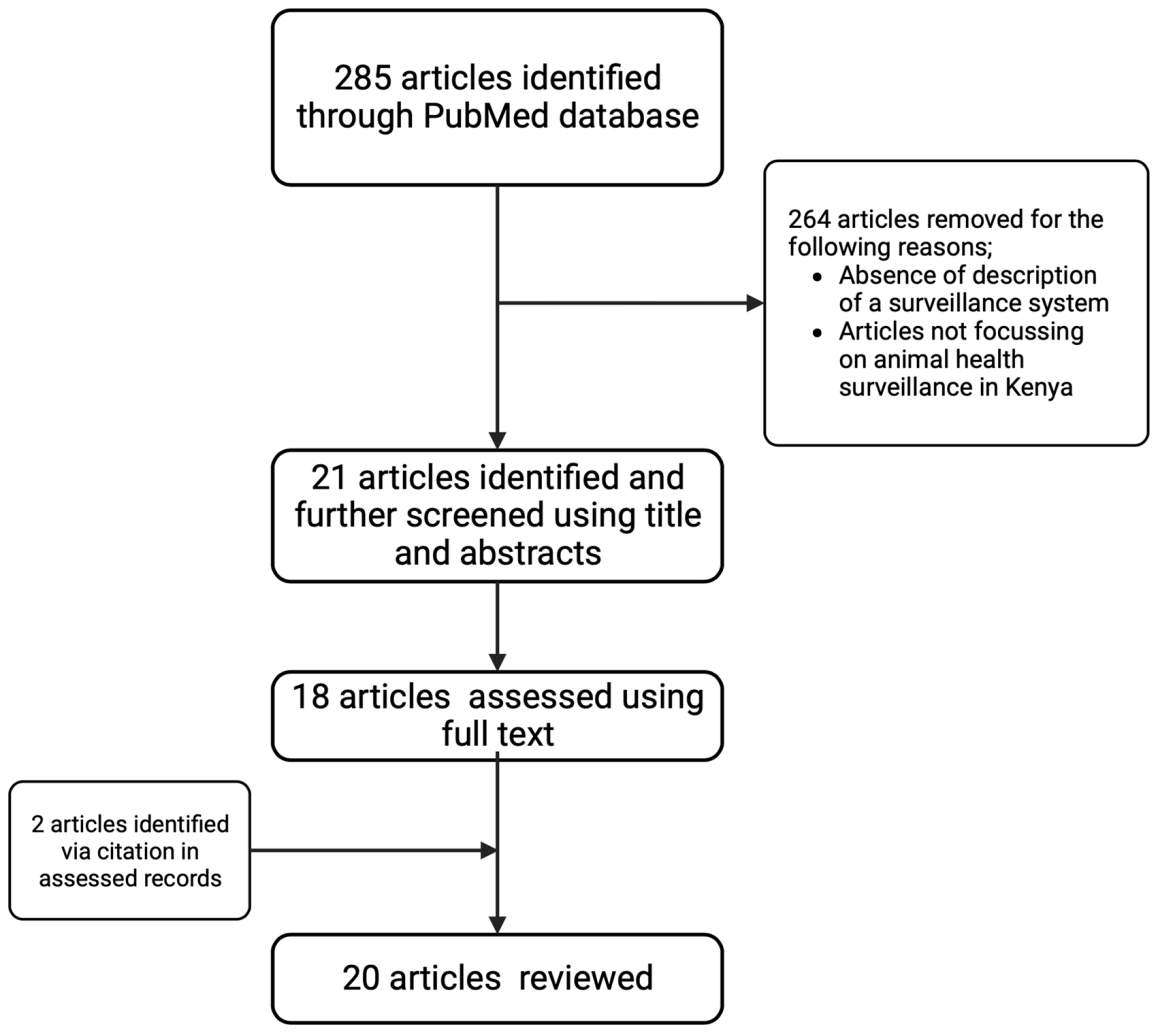
Flow chart describing the article selection criteria for the scoping review.

The Figure 2 outlines the process used in identifying the articles reviewed.

Six reports were identified from assessments and evaluations missions undertaken in Kenya in the last decade. These reports include the performance of Veterinary Services (PVS) by the World Organization for Animal Health (WOAH) in 2011, 2017 and 2022 (41, 42); the Joint External Evaluation (43); the assessment of animal disease surveillance capacity by the Food and Agriculture Organization (FAO) in 2017 (44); and the evaluation of surveillance systems relevant to zoonotic diseases in Kenya in 2015 (45).

### 3.1 Animal health surveillance in Kenya 1944-to date

#### 3.1.1 Evolution of the systems to date

Directorate of Veterinary Services (DVS) was established during colonial rule in 1895. It was among the first directorates to be created (21, 46). Around 1944, the European dominated areas of Colony and Protectorate of Kenya had begun to improve and diversify farming unlike the native Africans dominated areas where there were no efforts toward modernization since most pastoralists were seen to be less responsive to educational and missionary influence (21). The government during this time had a plan for compulsory dipping and immunization against certain priority diseases like rinderpest and rabies, introduction of improved animals, provision of sufficient dips and application of fencing ordinance. The government planned to improve native animal husbandry through propaganda, and education due to the anticipated slow adoption (21). Gene multiplication was undertaken in Ngong, Maseno, Baraton, Sangalo and Machakos which are currently Efficacy trial centers. During the same period a Central Artificial Insemination Station was established for semen production and distribution. The station would be supported by government until it become self-sustaining (21). Disease surveillance and diagnostic services in Kenya were governed by Diseases of animal Ordinance. The ordinance also guided application of quarantine rules and prophylaxis by vaccination and dipping as the key mechanisms for control of the diseases (21).

Between 1945 to 1958, total staff of veterinary department increased from 291 to 892 and headquarter grew from a small section to a separate ministry in 1954 and integrated on United Kingdom pattern in 1956 following the constitutional development of the colony. The ministry’s expenditure during the period was 10 per cent of the total government budget since the aim of the government was to maintain stable agriculture while conserving and developing land in accordance with the good husbandry practice.

Following insurrection of *Mau Mau* in 1952 to 1954, and subsequent detention of huge numbers of people in central province and surrounding areas, agriculture almost ran to a standstill as the government focused on restoring security until 1955 when Agriculture Ordinance organized markets and fixing of producer prices of major animal products leading to successful emergence of farmers from the *mau Mau* ordeal. Later in 1957, major decisions were made on animal husbandry and livestock improvement in African areas including establishment of District and provincial agricultural committees where African were members and allocation of different animal breeds to different regions in the colony and protectorate of Kenya (22).

The major diseases of concern during this time were FMD Type O and A and SAT 2 which appeared in 1957 in Samburu District among the African herds. FMD type C appeared in Machakos late 1957. The country required capacity for Research into FMD and this led to establishment of current FMD laboratory and vaccine production. Institute (Kenya Vaccine Production Institute - KEVEVAPI) in 1956 (22).

Veterinary services were offered as an essential service and the Director of Veterinary Service was an ex-official member of the parliament due to the crucial role played by the directorate (21). Before independence in 1963, clinical services were provided by private veterinarians with the Directorate of Veterinary Services (DVS) providing a regulatory role. Surveillance was predominately focused on notifiable diseases and used a passive structure, relying on reports from private sector veterinarians that served commercial ranches and dairy farms, being passed manually to the DVS (23, 46).

Shortly after independence in 1963, a decision was made to temporarily transfer the provision of clinical services to the public sector through the DVS. Plan to privatize veterinary services in Kenya commenced in 1976 with establishment of nine veterinary clinical stations (Tongaren, Karatina, Olkarau, Kericho, Sotik, Kakamega, Machakos, Thika, Nyahururu) (23) and six Regional Veterinary Investigative Laboratories (RVILs) were established between 1973 and 1987 (47) with a mandate to support veterinary diagnostic services in the field. These infrastructures were intended to gradually be privatized and become self-sustainable. During this period disease reports were in the form of narrative reports which generally lacked important epidemiological information, hindering epidemiological investigations and disease control efforts.

In 1981, the Veterinary Epidemiology and Economics Units (VEEU) was established with donor support and mandated to manage animal health data and disseminate relevant information (46). The VEEU developed enhanced tools to capture animal health events with sufficient spatial, temporal, and species data. These included the Notifiable Disease form (ND1) and Zero Report forms (18). In 1983, the government implemented a “District Focus” plan which established District treasuries to finance the operations at the district level. National level was left with the responsibility for general policy and planning of multi-district and national programs. This also shifted the district veterinarians financing model from Local Purchasing Orders at their disposal to an authority to incur expenditure with a budget ceiling (24).

The VEEU became dormant after the end of donor support around the mid-1980s other than a restricted remit supporting the Rinderpest eradication campaign (1987-2009) (26, 27). In 1988 the government dropped the ‘full employment policy for veterinary doctors’ which had previously provided public sector recruitment of veterinary graduates on an annual basis. Consequently, surveillance and disease control were negatively affected particularly in the Arid and Semi-Arid Lands (ASAL) where private veterinary practice were less viable (46). This preceded the full implementation of the donor-dictated Structural Adjustment Program (SAP) in 1990 (48) which decreased government involvement in the delivery of animal health services resulting in the collapse of most services including disease surveillance. This collapse and lack of government support to cushion private practitioners working in the ASAL regions may have contributed to the spread of several animal diseases across the country resulting in their endemic status (47).

In 2010 constitutional change led to the formation of 47 counties in 2013 with devolved powers (49). The division of responsibility between the two levels of government, was such that counties were mandated to provide county health services, including veterinary services while the national government provided national-level referral health services, disaster management, and formulating health and veterinary policies (50). A previous proposal by health professionals during the constitutional development stage to create a health service commission to secure the chain of command was rejected at the final stages of the constitutional review process and all services proposed under the commission, including veterinary services, were automatically placed under county health services (50). Notwithstanding the provision of the constitution, veterinary services remained domiciled in the Agriculture Departments and ministries even after the promulgation of the constitution. Following this, the direct reporting lines between the field surveillance officers and the DVS were hampered (45). While the DVS is the competent authority for veterinary services in Kenya, implementation of disease prevention control activities is a preserve of county governments, and this presented challenges in the coordination of disease control (45).

**Figure 3:**
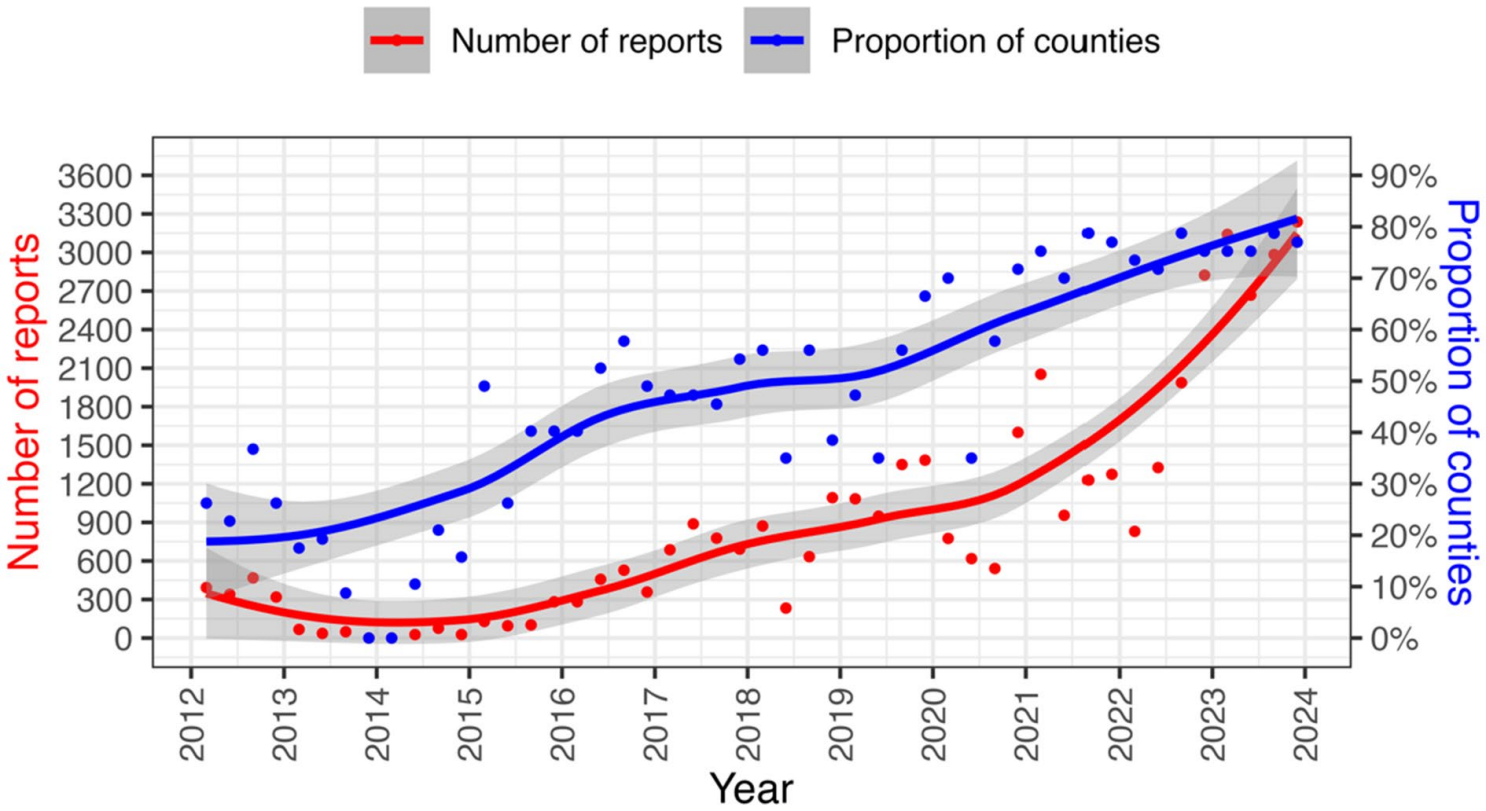
Animal diseases reporting trends from 2012 to 2023. Source DVS data.

Disease reporting to the DVS sharply declined after the promulgation of the Kenyan Constitution in 2010 with the biggest impact being felt in 2014 as shown in Figure 3. Consequently, the VEEU team sought collaborations with AU-IBAR through the Standards Methods and Procedures in Animal Health (SMP-AH) to hold a consultative meeting with key stakeholders from the counties on challenges affecting surveillance. Complex and unharmonized reporting systems, complex data collection tools, inadequate feedback from stakeholders, inadequate capacity building for technical personnel, and low prioritization for disease surveillance activities were reported as key issues associated with the decline (51).

Following this feedback, the VEEU team instituted corrective measures which included the creation of an email group (VETINFO) for data sharing, simplifying, and standardizing reporting tools and uploading the standardized tools into a harmonized mobile application (Epicollect), creation of WhatsApp groups and google group for communication and capacity building of technical personnel in counties on the electronic surveillance systems. The DVS also developed guidelines for the delivery of veterinary services under the devolved system. Though not legally binding, the guideline outlined obligations for the counties, especially on their responsibility to report animal diseases and related events to the Directorate of Veterinary Services to comply with international treaties ratified by Kenya on the application of sanitary and phytosanitary measures (34).

Efforts to streamline animal health surveillance in Kenya and adoption of technology in surveillance resulted in improved reporting performance from 2014 onwards as seen in Figure 3.

**Figure 4:**
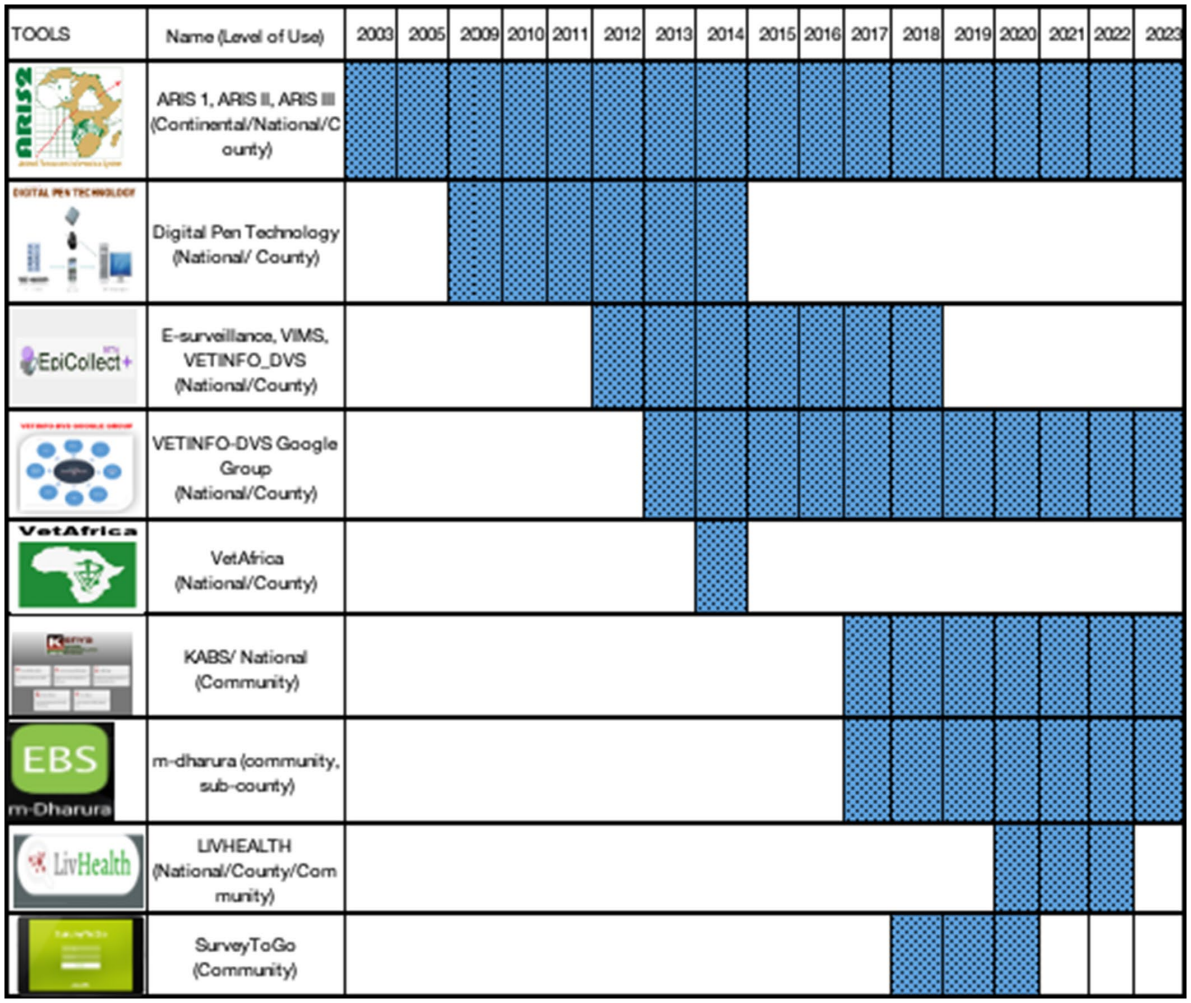
Evolution of e-surveillance tools in animal health in Kenya.

The Epicollect surveillance system, however, presented several limitations including data access issues resulting from the absence of a local server and a limited capacity for customization according to the needs of the country, and was therefore only used as a stop-gap measure. Previously, from 2003, Kenya piloted various electronic disease reporting tools including Digital Pen Technology, Epicollect (Veterinary Information Management System (VIMS), Epicollect plus, Epicollect Beta plus, and Epicollect 5), VETINFO-DVS google group, VetAfrica, and ODK collect (Liv Health). The evolution of electronic tools for animal Health surveillance in Kenya is outlined in Figure 4.

Epicollect was used as the official reporting tool for the DVS until the Kenya Animal Bio-surveillance System (KABS) was developed (52). This is a mobile-based technology that provides for the creation of forms for data collection, data analysis, and feedback. Data is stored in the DVS server for security. KABS introduced syndromic surveillance to the previously existing surveillance tools. The progressive roll-out of KABS commenced in 2017 and was taken up by all 47 counties by the end of 2021 and was confirmed to process capacity to enhance preparedness for epidemics of zoonotic diseases (19). During the same period, Kenya customized wildlife surveillance tools and incorporated them in the KABS to capture events in the wild populations (19). The tools were to be used by wildlife veterinarians, researchers, wardens, and others who interacted with sick wild animals. All the target users in the wildlife sector were also trained on the use of the system. However, only 18% (22/120) of the trained users submitted at least one report through the system from 2017 to 2021 (38). In 2017 mDharura was developed jointly by human and animal health sector and undergoing progressive roll out in counties to date. The system aims to support event-based surveillance where signals in both sectors are collected by members of community and shared across the sectors. The signals undergo verification and response depending on whether they are animal only, human only or zoonotic in nature. After verification, the event is reported through KABS or Kenya Health Information System (KHIS) as appropriate.

#### 3.1.2 Describing the flow of surveillance information in the current system

Here we describe the current functioning of the animal health surveillance system in terms of the flow of information through the system. Frontline animal health workers receive information from livestock keepers (passive surveillance) and undertake routine active surveillance at the villages, abattoirs, and livestock sale yards. In some counties where community disease reporting and event-based surveillance has been adopted (53) the Community Disease Reporters (CDRs) and/or Community Health Promoters (CHP) send information on the observed clinical signs or events which are verified by the frontline animal health workers, responded to where possible, and reported via KABS. The frontline animal health workers including private practitioners report disease occurrences using their mobile phones. The data in KABS is accessible to the sub-county and county administrators who can download data through the dashboard and use it to make their own disease control decisions. This information is also concurrently accessible to the VEES (the unit was changed to a section in 2021) (28). Currently AH surveillance in Kenya predominantly relies on passively collected clinical data with limited (7%) laboratory-diagnosed data. Additionally, the data also mainly (94%) comes from public sector practitioners, with only 6% coming from private practitioners (53).

The regional and national veterinary laboratories submit data to VEES every week through the VETINFO mailing group. Most laboratories use a standard Excel spreadsheet for reporting, while three of them utilize Laboratory Information Management Systems (LIMS). Data is downloaded in Excel format from LIMS, KABS, and the VETINFO group, then cleaned, and collated by a VEES epidemiologist. This process generates immediate notifications, quarterly feedback bulletins and monthly reports for international reporting to the World Organization for Animal Health (WOAH) and the African Union Inter African Bureau for Animal Resources (AU-IBAR).

The WOAH utilizes the World Animal Health Information System (WAHIS) to capture disease outbreaks (54). Regionally, the AU-IBAR uses the Animal Resource Information System (ARIS) which collects AH information from the member states monthly (55).

**Figure 5:**
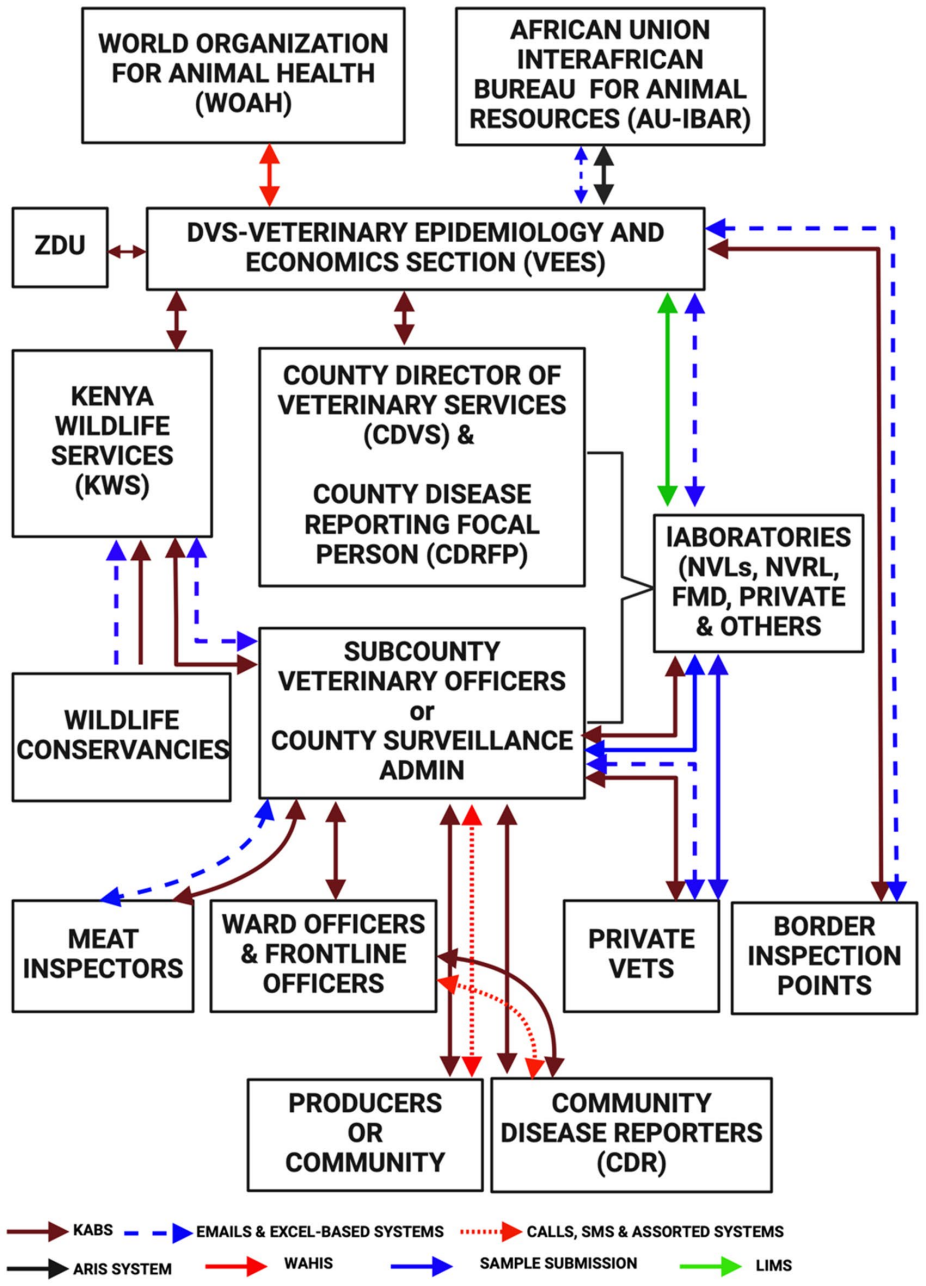
Flow of information and tools in the Kenyan Animal Health Surveillance systems.

The Zoonotic Disease Unit (ZDU) was established in March 2012, comprising officers from the Ministry of Health and the DVS. The unit was created to establish a framework for collaboration at the animal, human, and ecosystem interfaces for the management of zoonotic diseases (17). The unit has been instrumental in coordinating zoonotic disease response activities and officers in the unit have access rights to data in the KABS system. The data flow within the current AH surveillance systems is illustrated in Figure 5.

#### 3.1.3 Current diagnostic capacity linked to animal health surveillance in Kenya

Veterinary diagnostic services in Kenya are mainly a national government function under the DVS. The diagnostic services comprise six National Veterinary laboratories (NVLs) each serving a block of counties (56). There also exists reference laboratory services with the NVRL (National Veterinary Reference Laboratory at Kabete and the FMD Laboratory at Embakasi). A new Biosecurity Level Three (BSL3) Laboratory is also in the process of being established in the country. Various counties have also made attempts to establish basic laboratory testing to supplement the national laboratory network (42).

In March 2022, the National Reference Laboratory achieved ISO 17025 accreditation and proficiency testing which provides the DVS with the ability to certify animals and animal products per importing trading partner requirements as well as relevant international standards (57). The capacity of the veterinary diagnostic services is negatively affected by insufficient resources to conduct outbreak investigations and submit samples from suspected priority disease events, inadequate personnel, and inadequate supplies (42, 44). The structure of the veterinary laboratory system is currently not anchored in a formal legal instrument.

#### 3.1.4 Governance and funding of current animal health surveillance in Kenya

Kenya has a devolved system of government (50) which when properly implemented, the structure of administration brings decision-making closer to the actors thus allowing for the allocation of funds to meet the needs of local communities. Many success stories have been documented in some counties including the employment of adequate surveillance officers, and customized approaches to prevalent animal health events among others (45). On the other hand, the creation of semi-autonomous surveillance planning can present many difficulties. The lack of a direct reporting line from the CDVSs to the DVS presents a challenge in the coordination of the surveillance activities around the country leading to heterogeneous disease surveillance efforts between counties.

Surveillance activities often rely on resources that are also used for other animal health activities and in many cases, there are no resources specifically designated for surveillance (14). This lack of dedicated resources extends to the means of transport for surveillance teams as most counties do not have dedicated vehicles that can be used in emergencies (44, 45). Furthermore, in the event of an animal health-related emergency, government funding for surveillance and response is often delayed due to a lack of a proper contingency fund. This delay has the potential to hinder the effectiveness of the control program. As a result, there is a heavy reliance on project funding which compromises the long-term sustainability of the surveillance efforts.

### 3.2 Retrospective analysis of the surveillance system attributes

The data extracted from publications and reports regarding surveillance system attributes is summarized in the Table 1. The usefulness of the system has been experienced in some cases such as an enhanced syndromic surveillance to make decisions on RFV prevention and control (14). However, the system lacks a reliable early warning system which is due to limited resources (58). The usefulness of the system declined especially after devolution when it could not detect events of public health importance in a timely manner (45). The current surveillance system has been friendly, cost-effective and simple to use (19, 59). A previous evaluation also confirmed the flexibility of the system (60). On the contrary, the system was slow to adapt to devolution changes (45). To improve the acceptability of the system, there is a need to cultivate political goodwill and engage stakeholders as well as provision of timely feedback that can inform local decision-making (14, 61). The positive predictive value of the system is negatively affected by the minimal utilization of the laboratory in diagnosis due to the costs involved and sometimes inadequate capacity of the available laboratories (41, 43). The system was representative as it used different data sources and different systems such as syndromic surveillance, sentinel surveillance and adopted the use of a real time electronic reporting system which improved reporting rates and spatial distribution of reports. However, this is negated by reporting gaps experienced (43, 45, 62), weak active surveillance (41, 44, 74), and inadequate involvement of private practitioners and the community in surveillance (43, 44, 60, 66). Timeliness of the system received a major boost following the introduction of syndromic surveillance and a mobile-based electronic reporting system (19, 58, 69). Collaborations in the system are enhanced by presence of ZDU platform (44, 71) but there is a weak intra and inter-county collaboration as well as collaboration between the public and private sectors (44).

**Table 1:** Summary of the surveillance system attributes for the animal health surveillance systems currently active in Kenya articles identified and the key findings with effect on surveillance.

Attribute	Importance to EWS	Indicator	Key Findings and Citations
Usefulness	Enables evidence based and timely response to events	Decisions made from surveillance information	Lack of formal operational structures and poor allocation of resources to disease surveillance leading to weak early warning system thus reduced usefulness (14). An enhanced syndromic surveillance system implemented during El Nino against RVF improved early warning system and was successfully used to make decisions (58). The system was not able to detect diseases or adverse exposures of public health importance in a timely manner, especially after the devolution of animal health services to the county governments (45).
Simplicity	Ease of learning and understanding the system enabling adoption and utility	Staff training requirements, number, and type of reports	Numerous electronic animal disease-reporting systems have been piloted in Kenya, but most have not been implemented due cost, lack user-friendliness, and data insecurity (19). 100% of the users of KABS in Narok County in Kenya reported that it was easy to operate the mobile application (59)
Flexibility	Allows adaptation to changing information needs, e.g., new diseases, change in case definitions, etc.	Evidence of response to new demand	In an evaluation carried out in Narok County, the current electronic surveillance system is easy to accommodate proposed changes indicating flexibility (60). The system was slow in adapting to changes like the devolution of veterinary services to the counties (45).
Acceptability	Increased acceptability improves the sensitivity of the system thus enhances rapid detection and response.	Participation rate and how quick that was achieved, timeliness and report completeness	Inadequate political good will affect the overall acceptability and should therefore be cultivated (14). Importance of engaging with local stakeholders in the field, while also providing timely feedback through public engagement sessions, to ensure ongoing compliance and acceptability at all levels (61).
Positive Predictive Value	A surveillance system with a low positive predictive value has more false positives and may lead to misallocation of resources.	Laboratory confirmation of cases.	Limited utilization of laboratory services (38, 45) and Inadequate capacity in specimen referral system for the animal health sector at the sub-county level (41, 43). Presence of a laboratory network that supports diagnosis for priority diseases (44). Presence of point of care test for some disease (43).
Representativeness	Accurate description of a health event and improved sensitivity	Data quality, data sources, collaborations	Only low levels of active surveillance have been applied since 1964 while surveillance is mainly passive and relies on outbreak reports (41, 62). There exist gaps in historical records affecting data quality (62). There is an underestimation of some infections by some pathogens like *Coxiella burnetti* and *l*ack of active surveillance on some diseases/pathogens like Q fever and their control efforts thus the need to explore integrated disease surveillance and prevention/control programs in Kenya (63). Low disease reporting rates (43, 45). An enhanced syndromic surveillance system implemented during El Nino against RVF improved early warning system and acted as an excellent pilot for designing and implementing syndromic surveillance in animals in the country (58). Surveillance by sampling animals at slaughter and tracing movement through participatory methods can improve active surveillance against zoonoses like RVF (64). The introduction of electronic reporting system resulted in a 2- to 14-fold increase in number of disease reports and spatial distribution (19). The need to have information from all parts and all geographical areas of the country was underscored by finding that periodic expansion of vectors may occur in presence of RVF susceptible animals and the transmission occurrence during the interepidemic periods (65). Involvement of community and private practitioners in improving early warning systems should be improved in Kenya (43, 44, 46, 59, 66–68). Sentinel herds surveillance exists for HPAI and RVF (44).
Timeliness	Enhances rapid detection and response	Availability of information for immediate disease control interventions	Presence of enhanced syndromic surveillance system improves early warning system and timeliness in detection of cases (58). Digital bio surveillance for zoonotic diseases including use of local news and socio media improves timeliness and should be adopted in Kenya to improve EWS (19) (69). Prompt prediction of disease outbreaks would thus enable early interventions that would reduce morbidity, mortality, and general economic losses (70).
Collaborations	Fosters coordination and timely joint response to events of public health potential	Evidence of a formal mechanism of coordination or structure	There exist some collaborations between human and animal disease surveillance officers at the sub-national level, driven by common objectives such as meat hygiene and response to suspected rabies and anthrax cases (14). Lack of formal mechanisms for timely information sharing between animal, human (43). Establishment of a framework for multi-sectoral collaboration Zoonotic Disease Unit (ZDU) However, implementation at subnational administrative levels, sustainability, competing priorities, and funding deficiencies remain as challenges (44, 71) Kenya prioritized zoonotic diseases in a collaborative approach (72). Epidemiological investigation of a Rift Valley Fever outbreak in humans and livestock in Kenya undertaken collaboratively (73). Collaboration between the KWS and DVS is limited, with field-level coordination efforts hampered by low numbers of KWS veterinary staff (44) Intra- and inter-county collaborations not formalized (44). .Low involvement of private sector actors in surveillance (41)

### 3.3 Assessment of strengths, weaknesses, and recommendations from recent external evaluations

The animal health surveillance systems in Kenya have undergone various evaluations each providing key insights into their strengths and weaknesses (14–18). These evaluations form a good basis for prioritizing interventions to improve the surveillance systems.

Table 2 outlines all the identified strengths, weaknesses, and recommendations highlighted in the evaluation exercises for the animal health surveillance systems in Kenya over the past 10 years.

**Table 2:** Assessment of the key strengths, shortcomings, and recommendations as outlined in the recent evaluations of the animal health surveillance system.

Evaluation/Assessment mission	Key strengths identified in the systems	Key weaknesses of the systems	Recommendations for improvement	Reference
PVS Gap analysis mission, Kenya 2022.	Presence of a Surveillance Manual Comprehensive contingency plans are in place for rinderpest, RVF, FMD and Highly Pathogenic Avian Influenza (HPAI). Surveillance plan for antimicrobial resistance in place.	Insufficient vehicles to conduct surveillance for the county veterinary services and regional laboratories. Lack of operational funding affecting surveillance, follow-up on suspect outbreaks and movement control. Sub optimal personnel levels at the veterinary laboratories Absence of a structured risk analysis process. Surveillance mainly responsive due to inadequate resources.	Implement an early warning system. Optimize the number of staff at Border points to enhance surveillance and rumor reporting. Digitalize the storage, retrieval and data analysis and integrate KABS and LIMS. Strengthen awareness of animal owners, private veterinarians, meat inspectors, and live animal market workers to on reporting. Strengthen active surveillance for priority diseases	(75)
Joint External Evaluation of IHR core capacities of the republic of Kenya, 2017	Legislative mechanisms exist and several laws reviewed. Formal intersectoral coordination mechanisms between human and animal health exist, including the Zoonotic Disease Unit (ZDU) and the National Task Force Committee. Secondly, informal exchanges of information between ministries exist, based on personal contacts and goodwill. Experience in investigating and reporting public health emergencies to WOAH Presence of a Field Epidemiology and Laboratory Training Programme (FELTP) which trained epidemiologists in human and animal health. Inclusion of One Health curricula in veterinary and public health schools Point-of-care testing does occur for some diseases of public health importance. General syndromic surveillance and sentinel surveillance exist at four sites for emerging and re-emerging diseases such as Rift Valley fever. A community-based surveillance system is being piloted in selected counties. Mobile phone event-based surveillance in place Multisectoral human resources are available at a national level for both animal and human health.	Lack of formal mechanisms for timely information sharing between animal, human, and other relevant sectors, including surveillance and laboratory data. Inadequate capacity for a timely response to foodborne reports and events especially at county and sub-county (district) levels. Inadequate capacity in specimen referral system for the animal health sector at the sub-county level. Lack of integration of laboratory data into surveillance and reporting systems Low disease reporting rates in the animal health sector. Laboratory diagnosis of some priority zoonotic diseases is still inadequate, especially at subnational levels. Overreliance on donor funding to fund most aspects on the surveillance systems.	Establish formalized mechanisms for regular data sharing and information exchange between relevant sectors and stakeholders regarding public health events, using a One Health approach. Establishment of a centralized laboratory surveillance reporting system also covering the data from the public health and veterinary sectors. Full implementation of the National AMR surveillance system in the animal health sector Develop national control strategies for anthrax and brucellosis. Identify ways to encourage reporting at the county and sub-county levels in the animal health sector. Improvement of animal health workforce to population ratio especially in remote, arid areas Improvement of capacity for a timely response to zoonotic disease outbreaks Work on the sustainability of kits and reagents for both human and veterinary laboratories. Develop and implement the point-of-care testing guidelines. Integrate laboratory data into both the human and animal indicator-based surveillance systems. Enhance event- and community-based surveillance in both the human and animal health sectors. Strengthen the use of surveillance data for planning, advocacy, and early response. There is a need for automated data analysis	(43)
Assessing animal disease surveillance capacities, using Surveillance Evaluation Tool (SET) FAO 2017	Several legislations are in place to guide animal disease surveillance in Kenya. Specific technical workgroups in place include: One Health, rabies and RVF control, antimicrobial resistance (AMR) Descriptive statistics are regularly performed, and a quarterly bulletin is shared with the stakeholders. Presence of surveillance and response strategies and plans for some priority diseases. Animal disease surveillance plan under development. Presence of a laboratory network that supports diagnosis for priority diseases. Sentinel herds surveillance exists for HPAI and RVF A laboratory information management system (LIMS) is implemented at the CVL, and is being rolled out in the other regional laboratories. The establishment of ZDU and prioritization of the zoonotic diseases in Kenya was done in 2015. use of low-maintenance technology (WhatsApp and Google Group) that has improved the speed and efficiency of information sharing.	No formal steering committee or overarching technical committee exists within the system. Data collectors at the field level include community animal health workers (CAHW) and community disease reporters (CDR), which are largely supported by projects from external donors – their national distribution may be uneven based on these specific projects. No specific budgetary line for animal disease surveillance at the national level and each county is expected to factor in the respective budgets. Lack of direct supervision between the county veterinary services and the DVS Presence of many electronic systems pilots. Lack of standard operating procedures (SOPs) that specifically outline activities related to field investigations and data management. Some diseases lack formalized case definitions. Active surveillance and sentinel herd monitoring are affected by limited resources. Inadequate skills, Limited staffing, and laboratory supplies curtail the capacity of the laboratories. Collaboration between the KWS and DVS is limited, with field-level coordination efforts hampered by low numbers of KWS veterinary staff. Irregular staffing and reporting rates between counties leading to unrepresentativeness. Lack of a formal communication strategy Intra- and inter-county collaborations not formalized. Limited feedback to the field actors	Explore ways of making most of the private animal health service providers able to contribute to surveillance. Hold regular meetings with central and county representatives to improve coordination. Formalize National Surveillance Plan to update reporting legislation and facilitate lobbying of surveillance-specific funding stream. Institute a communication strategy Adoption of a single harmonized reporting system. Establish county epidemiology units. Develop a national laboratory strategic plan/network to coordinate the work of the different laboratories in the country and increase the effectiveness of current facilities	(44)
WOAH PVS Gap Analysis Report 2011	The veterinary services (*VS*) conduct passive surveillance for some relevant diseases and can produce national reports on some diseases. The *VS* conducts active surveillance for some relevant diseases (of economic and zoonotic importance) Kenya has a list of priority animal diseases and notifiable diseases.	Lack of an integrated surveillance system Low awareness among the stakeholders on surveillance Low involvement of private sector actors in surveillance Inadequate lab supplies and capacity. Active surveillance is only in a part of susceptible populations and is not regularly updated. Lack of SOP for active surveillance and laboratory procedures Lack of formal collaboration among the collaborators Lack of the necessary legal and financial support to respond to sanitary emergencies appropriately. Weak enforcement of disease control interventions	Build passive surveillance capacity at the DVO level for relevant diseases. Establish an integrated disease reporting system. Improve stakeholder awareness regarding surveillance and establish a feedback mechanism. Strengthen public-private partnerships in disease surveillance. Provide the necessary laboratory supplies. Active surveillance protocols should be designed and implemented per the relevant OIE Code provisions. Establish formal linkages with sectors having sanitary data. Design and implement an animal identification system to achieve animal traceability per Chapter 4.2 of the OIE Code. Provide detailed SOPs for active surveillance (based on OIE Standards) to be implemented, including detailed laboratory procedures and methodology for result interpretation. Formalize and intensify collaboration with other relevant government organizations. Provide SOPs for timely decision-making processes for identified sanitary emergencies	(41)
OIE PVS Veterinary Legislation Identification Mission	Since the previous PVS Evaluation Follow-Up mission in 2011, advanced significantly in enhancing passive surveillance activities in Kenya. There are selective active surveillance activities such as sentinel herd surveillance for Rift Valley Fever (RVF), Peste des Petits Ruminants (PPR) and Contagious Bovine Pleuro-Pneumonia (CBPP), and vector surveillance for Trypanosomiasis. Comprehensive contingency plans are in place for rinderpest, RVF, FMD, and Highly Pathogenic Avian Influenza (HPAI).	lack of resources and funds, surveillance leads to only “responsive surveillance” i.e. sample collection in the event of suspect outbreaks for which a laboratory diagnosis is required. No routine national active surveillance programs in place. Implementation of the contingency plans is however hampered by lack of resources and funds. Shortage of suitably qualified and experienced veterinarians, especially at County headquarters and sub-sub-county In several Counties there is political interference and potential loss of technical authority of the Director of County DVS, with technical decisions challenged in some Counties. Some laboratory equipment are obsolete. Lack of a structured risk analysis process to facilitate the decision-making process to prevent the introduction and spread of disease	Review staffing needs at County level in accordance with functional and operational needs. Further deployment of Community-Based Animal Health Workers (CBAHW’s) in the ASAL areas to compliment the shortage of qualified veterinary paraprofessionals in these areas. Legislation reviews to enable registration of CBAHWs, accommodate devolution, delegation to private practitioners, define national standards and technical integrity and independence. Develop detailed annual investment and action plans. Establish effective risk analysis and risk management at DVS Kabete and train CDVS for implementation of identified mitigation measures. Continue with the roll out of the electronic reporting tools. develop and implement comprehensive annual communication plans to ensure that all stakeholders are kept informed	(42)
Evaluation of Surveillance Systems Relevant to Zoonotic Diseases in Kenya-2015	Disease surveillance activities are supported by veterinary laboratories in the country. Feedback available on quarterly basis through quarterly bulletin The system was able to detect trends that signal changes in the occurrence of diseases including detection of epidemics or outbreaks. Presence of a Standard Operating Procedure (SOP) for surveillance activities	Passive surveillance is faced with challenges of underreporting. Multiple disease reporting tools available. All digital reporting tools were less than 50% the rest were paper based tools. The system was not integrated to other surveillance systems leading to low uptake. The system was not able to detect diseases or adverse exposures of public health importance in a timely manner, especially after the devolution of animal health services to the county governments. The system was not able to estimate the magnitude of morbidity and mortality related to the health-related event under surveillance due to lack of representativeness. The system was slow in adapting to changes like the devolution of veterinary services to the counties. Use of standard case definition and laboratory diagnosis was minimal leading to generation of data that may not be factual.	Integrate the various aspects of the system so that single output is generated from the various systems. Harmonization of the tools, development of a strategic plan for electronic reporting and development of Standard Operating Procedures (SOP’s) for each system Establishment of an integrated system	(45)

Key strengths identified include: a strong legislative framework for disease surveillance and control (41–44), a formal intersectoral coordination mechanism between the human and animal health sectors (43–45), presence of adequate experience in investigating and reporting public health emergencies to WOAH, WHO,AU-IBAR, FAO and Africa CDC, provision of field epidemiology trainings to build local capacities, and inclusion of one health in school curricula (43). Other strengths include the availability of a laboratory network to support surveillance (43–45), syndromic and sentinel surveillance for emerging and re-emerging diseases (41–44), community disease reporting (43), electronic disease reporting systems and LIMS (43–45), comprehensive contingency plans, specific disease control strategies as well as use of low maintenance technological initiatives like WhatsApp and Google groups for information sharing (41–45) (44). The is also feedbacking mechanism through a quarterly bulletin shared with field actors and stakeholders (45), as well as considerable capacity for data analysis (44). The PVS follow-up evaluation indicated significant improvement in passive surveillance following recommendations from previous evaluations (42), underscoring the importance of regular evaluations.

The evaluations also identified several shortcomings and gaps within the surveillance systems. These include the absence of a formal mechanism for data sharing across collaborating sectors due to the lack of a legal framework for ZDU establishment (41, 43), inadequate capacity for timely response to reported events due to insufficient funding for surveillance (42–44), insufficient diagnostic capacity due to unreliable supplies, low staffing, obsolete equipment and inadequate skills (42, 43), lack of integration of surveillance systems across sectors including laboratory systems (41, 43, 45), low disease reporting rates in the country leading to unrepresentative data (43–45), limited utilization of diagnosis (43), inadequate involvement of private sector actors and the community in disease surveillance (41, 42, 44), limited use of rapid diagnostic kits to support detection (43), lack of a direct supervisory role between the DVS and CDVS causing complications in coordinating surveillance activities (44), and potential political interference threatening the technical authority of the CDVSs (42). Most evaluations also acknowledged weaknesses in active surveillance which largely relied on the availability of resources and partner organization activities (41, 42, 44). Lack of stakeholder awareness, weak implementation of strategies and enforcement of laws, and the lack of standard case definitions for priority diseases were also key shortcomings hindering the capacity of the animal health surveillance systems in Kenya (41–44). Furthermore, the surveillance systems faced challenges such as staff shortages and a lack of a structured risk analysis process to facilitate decision-making and the implementation of preventive and control measures (42, 44).

## 4 Discussion: utility of animal health surveillance in Kenya for efficient detection of zoonotic diseases

The animal health surveillance in Kenya has evolved and experienced growth and challenges over the last 80 years. Major changes occurred during key periods such as pre-independence, the independence period, government programs like rinderpest eradication, the structural adjustment program around 1990, devolution of veterinary services and the roll out of the electronic reporting systems in the country. This study provides a summary of these experiences, highlights identified gaps and aims to improve the systems for integrated surveillance systems and a public health early warning system.

Surveillance approaches for early warning are complemented by effective laboratory testing to diagnose the underlying infectious causes of emerging trends and alarms (76). However, accurate diagnosis of animal health events in Kenya is greatly hindered by the sparse distribution and limited capacities of veterinary laboratories (31, 45). Field surveillance officers bear the cost of sample collection and submission leading to a majority opting for clinical diagnosis. This compromises data quality and the positive predictive value of the surveillance system (45).

The current national surveillance may not accurately reflect the true burden of diseases in the country. Consequently, mapping disease risk using this surveillance data may be challenging and less accurate. This is due to low representativeness of the data which arises from inadequate participation by some stakeholders and low reporting rates (20). After the privatization of veterinary services, most of the sick animals are now treated by private practitioners (14) and veterinary medicine shops (5). However, contrary to this, the private sector only contributes only about 6% of the data in the current animal health surveillance systems (53). To improve reporting rates, it is crucial to involve all stakeholders in surveillance. Although the involvement of the community in disease reporting has gained traction in the country, it is still inadequate. Only 10% of the counties have made attempts to roll out (53) community disease reporting. Previous studies have emphasized the importance of community information and ongoing surveillance in EWS (59, 67). However, the adoption and utilization of wildlife surveillance tools in KABS has been lacking, leading to minimal complementarity from other data sources (5). This lack of adoption may be attributed to the possible requirement for wildlife veterinarians to send reports in other different templates, leading to duplication and user fatigue. Therefore, continuous capacity building and awareness creation are needed in this area. In addition to current data sources, it is important to consider other sources such as livestock producers, livestock markets, abattoirs, zoo-sanitary checkpoints, dips/ crushes, veterinary laboratories, and veterinary medicine shops (77). These sources can provide valuable data for disease surveillance and important components in the big data for surveillance.

Currently surveillance experts are concerned with multivariate surveillance systems which entails monitoring multiple variables and indicators from different data sources. This increases the probability of detecting important events as a single data source may miss crucial aspects of an outbreak (66, 77). The current systems, which mainly focus on a univariate approach, should adopt the application of big data, including syndromic surveillance data, community disease reports, production data, wildlife surveillance data, climatic data, animal treatment records, livestock identification data and socio-economic factors coupled with Artificial Intelligence (AI) as demonstrated in previous studies to improve efficiency in the management of animal health and zoonotic related risks (78, 79). This will enable the surveillance system to provide reliable information from complex analytical models for decision making thus partially mitigating effects of inadequate staffing and analytical capacity throughout the country.

Active surveillance for most priority diseases in Kenya has been happening at very low levels since independence (62). Most active surveillance activities mainly depend on donor-funded projects or occur on need basis (41, 42, 44). However, incorporating sentinels into surveillance systems has increased the likelihood of detecting of the first incursion of a particular disease in the shortest time possible (76). Therefore, utilizing early warning systems, such as sentinel surveillance in vectors, wildlife, companion animals, and zoological parks, has been recognized as the key method for improving surveillance of emerging diseases (80) and could be strengthened in Kenya. Participatory surveillance is also an active surveillance approach that can result in enhanced collaboration and communication among different sectors and institutions. This can help better understand the causes of diseases, determine the success or failure of surveillance programs, contribute to policy reforms, or provide a quick overview of the epidemiological situation in an area (76). Active surveillance at abattoirs could also leverage livestock movement and employ participatory methods to improve active surveillance for zoonotic diseases like Rift Valley Fever (64). The increasing use of electronic data collection and electronic data interchange by surveillance systems promotes timeliness and increases the usefulness of reporting (20). Mobile-based surveillance systems are known to capture higher numbers of AH events compared to traditional surveillance systems (68, 81). This is evident from the progressive improvement in reporting rates following the progressive roll-out of KABS, as seen in Figure 3. The real-time nature of the mobile technologies allows for constant update thus improving EWS for rapid response. The roll out of electronic reporting tools should leverage on the widespread use of mobile phones in sub-Saharan Africa, which is estimated to be 67% (82). Therefore, use of mobile technologies is prerequisite for efficient EWS. However, for the system to be efficient the key capabilities to consider include sustainability in resource-limited environments, a mobile application for field data collection, have local capacity for maintenance and capacity building, integration with laboratory and human health surveillance systems and the ability to analyze and import data from other sources.

Currently in Kenya, there is lack of integration between the epidemiological data collections system (KABS) and LIMS. As a result, duplication of work and complex management of data from various sources are occurring. To effectively protect public health, trade, and animal health and welfare its crucial to establish data sharing and collaboration mechanisms (63, 83). This will create synergies among relevant sectors and facilitate efficient management of EID EWS (17, 84) particularly in resource-limited countries like Kenya (14). The surveillance systems should be integrated with other health information systems allowing for data exchange and sharing in multiple formats, as well as data transformation. By doing so, individual systems can meet specific data collection needs without duplicating effort or causing disharmony in data management (20).

There is a need for continuous review and updating of the existing legislation that governs the surveillance systems in Kenya to create a common objective and to enhance structured collaborations among stakeholders (14). Therefore, constant advocacy and review of legislation are key for OH’s approach toward improving the sustainability of EWS. This should be coupled with modern innovative mechanisms for data sharing.

External evaluations are key in identifying the key intervention areas for improving of the surveillance system. Due to the availability of many surveillance evaluation tools or guidelines, Kenya should develop and implement an internal surveillance evaluation guideline with country-specific indicators. To operate optimally, these systems should be regularly monitored and evaluated for continuous improvement. The surveillance and Information Sharing Operational Tool (SIS-OT) could also be considered to identify gaps in multisectoral surveillance and information sharing for zoonotic diseases for improvement (85).

The provision of veterinary services as a private good especially in ASALs where there are limited numbers of private practitioners may not be feasible. Therefore, animal disease surveillance and control activities should be offered as a public good to minimize the spread of animal diseases.

## 5 Conclusion

In conclusion, the animal health surveillance systems in Kenya have evolved over time. This study provides valuable insights into the strengths, weaknesses, and opportunities of the system at different stages of development. These insights can be used to improve the current early warning systems for rapid disease detection and response. Several potential areas of improvement have been identified, including adopting of harmonized reporting tools, establishing a clear chain of command across all levels of government, implementing electronic-based surveillance systems, integrating Artificial Intelligence in surveillance, developing of case definitions for priority diseases, incentivizing disease reporting, regularly evaluating the systems, involving the private sector players, wildlife and community in surveillance, strengthening legal framework for collaboration mechanisms, and providing regular support.

However, it is important to acknowledge the limitations of this study. It primarily relied on secondary data which may have resulted to overlooking certain aspects of the surveillance system evaluation. Therefore, further studies are recommended to comprehensively evaluate the current systems. The use of tools like the Surveillance and Information Sharing Operational Tool (SIS-OT) is recommended for this purpose.

In summary, this research contributes to the advancement of animal health surveillance in Kenya by highlighting the areas that require the attention from the government. By addressing the identified shortcomings, the effectiveness and resilience of animal health surveillance in Kenya can be enhanced, leading to improved timely disease detection, response, and control.

## Data availability statement

The raw data supporting the conclusions of this article will be made available by the authors, without undue reservation.

## Ethics statement

The requirement of ethical approval was waived by ILRI Institutional Review and Ethics Committee for the studies involving animals because the study used retrospective data mainly and did not involve primary data collection and sample collection. However the use of the data was approved by the Directorate of Veterinary Services. The studies were conducted in accordance with the local legislation and institutional requirements.

## Author contributions

SK: Supervision, Software, Resources, Funding acquisition, Writing – review & editing, Writing – original draft, Visualization, Validation, Project administration, Methodology, Investigation, Formal analysis, Data curation, Conceptualization. ST: Writing – review & editing, Writing – original draft, Visualization, Validation, Supervision, Software, Resources, Project administration, Methodology, Investigation, Funding acquisition, Formal analysis, Data curation, Conceptualization. BB: Writing – review & editing, Supervision, Resources, Project administration, Methodology, Investigation, Funding acquisition, Data curation. MWM: Project administration, Investigation, Writing – review & editing, Validation, Supervision, Methodology. NN: Writing – review & editing, Methodology. AO: Writing – review & editing, Methodology. MM: Writing – review & editing, Methodology. LT: Writing – review & editing, Writing – original draft, Visualization, Validation, Supervision, Software, Resources, Project administration, Methodology, Investigation, Funding acquisition, Formal analysis, Data curation, Conceptualization.
